# A Case of Duffy‐Null‐Associated Neutropenia in a Patient With Chronic Myeloid Leukemia

**DOI:** 10.1155/crh/9335086

**Published:** 2026-09-26

**Authors:** Anushka Parekh, Joanna Metzner-Sadurski

**Affiliations:** ^1^ Edward Via College of Osteopathic Medicine-Carolinas Campus, Spartanburg, South Carolina, USA; ^2^ Self Regional Healthcare, Greenwood, South Carolina, USA

## Abstract

Duffy‐null‐associated neutrophil count (DANC) refers to a below‐normal absolute neutrophil count (ANC < 1.5 × 10^3^/L), present most commonly in African American individuals. Neutropenia is a result of a lack of Fya and Fyb antigens on red blood cells. These patients do not have an increased risk of infection and may have an ANC < 1.5 × 10^3^/L at baseline. This unique case presents Duffy‐null‐associated neutropenia in the setting of chronic myeloid leukemia (CML). A 64‐year‐old African American female presented to the emergency department on May 17, 2021, for fatigue and was found to have a white blood cell (WBC) count of 65.3 × 10^9^/L. Hematology and oncology referral led to a diagnosis of CML. Subsequently, she began treatment with imatinib (Gleevec) 400 mg daily. One month into treatment, she was neutropenic, with an ANC of 0.6 × 10^3^/L. For the next several years, this patient repeatedly had treatment holds and dose reductions in response to recurrent neutropenia. When BCR‐ABL1 transcript level continued to increase, indicating poor treatment response, she was referred to Medical University of South Carolina (MUSC). After several treatment changes due to adverse effects, she began treatment with asciminib 80 mg daily. BCR‐ABL1 transcript level with asciminib was consistently below 0.1%, indicating successful treatment response. Despite this milestone, she continued to have episodes of neutropenia, prompting a check for Duffy antigen presence. Testing confirmed the lack of Fya and Fyb antigens, indicating Duffy‐null status. This case demonstrates the importance of identifying Duffy‐null individuals. Despite achieving major molecular response, the patient had persistent neutropenia, leading to the investigation of Duffy‐null status. It is hypothesized that underlying Duffy‐null status may have caused neutropenia, rather than an adverse reaction to treatment. Early detection of Duffy‐null status can prevent dose reductions in therapy and delayed remission. Particularly with African American patients, a better framework for addressing Duffy‐null neutropenia may benefit long‐term outcomes in the setting of cancer therapy.

## 1. Introduction

Duffy‐null‐associated neutrophil count (DANC) refers to a below‐normal absolute neutrophil count (ANC), present most commonly in African American individuals. In the United States, approximately two out of three people who identify as black are Duffy‐null [1]. The Duffy blood group proteins are produced by the FY gene located on chromosome 1. There are two primary Duffy antigens, Fya and Fyb [1]. Duffy‐null patients lack the Fya and Fyb antigens on red blood cells. Fascinatingly, this provides Duffy‐null individuals resistance to *Plasmodium vivax*, the primary causative agent of Malaria [2]. This phenomenon is a result of evolution which correlates to the geographic area in which Malaria prospers [2].

Individuals that have the Duffy‐null phenotype are known to be at risk for neutropenia [1]. This variation of neutropenia is linked to the Duffy antigen receptor of chemokines (DARC) also known as atypical chemokine receptor‐1 (ACKR1), giving it the name DANC [3]. The mechanism is not thoroughly understood; however, it is hypothesized that since ACKR1 functions to regulate chemokines, therefore mediating neutrophil migration, the lack of this receptor may be the cause for neutropenia in these patients [1].

Neutropenia is defined as an ANC of < 1.5 cells/μL in adults [4]. While neutropenia typically means an increased infection risk, this is not the case in Duffy‐null individuals. For this reason, DANC was historically referred to as benign ethnic neutropenia. These patients in fact do not have an increased risk of infection and may have an ANC < 1.5 cells/μL at baseline [4].

This case presents a unique case of Duffy‐null‐associated neutropenia in the setting of chronic myeloid leukemia (CML). CML is an abnormal proliferation of myeloid cells, most commonly due to the BCR‐ABL1 fusion gene on the Philadelphia (Ph) chromosome [5]. Now finally in remission on a third‐line tyrosine kinase inhibitor (TKI), this patient had persistent neutropenia from an unknown cause, causing disruptions and dose reductions in her treatment. Finally, she was identified as Duffy‐null, providing an explanation for her neutropenia. The wide prevalence of Duffy‐null individuals suggests that more awareness and testing should be conducted in patients with persistent asymptomatic neutropenia. Specifically in cancer treatment, dosing and therapy commonly rely on a sufficient ANC given the risk of infection and immunosuppression. Identifying Duffy‐null individuals can reduce therapy interruptions by reconsidering the ANC barrier and establishing a unique reference range for these individuals.

## 2. Case Description

A 64‐year‐old female with a past medical history of hypothyroidism, diverticulosis, gastroesophageal reflux disease, and hypertension presented to the emergency department on May 17, 2021, for fatigue and was found to have a white blood cell (WBC) count of 65.3 × 10^9^/L. Hematology and oncology referral led to a thorough workup including repeat basic labs, peripheral blood smear, flow cytometry, RT‐PCR, and BCR‐ABL1 transcript level. Peripheral blood smear was remarkable for increased WBC, neutrophilia with left shift, and basophilia. Flow cytometry revealed 3% blasts, granulocytosis, and basophilia. P210 BCR‐ABL1 was detected upon RT‐PCR; however, JAK2, CALR, and MPL mutations were undetectable. The BCR‐ABL1 transcript level was found to be 68.75%. Contradictory to her presentation, this patient did not have palpable splenomegaly. Ultrasound of the abdomen revealed splenic length of 9.5 cm, which is within normal limits. These findings led to a diagnosis of CML. Bone marrow aspiration was not performed at this time.

Following diagnosis, she began treatment with imatinib (Gleevec) 400 mg daily. One month into treatment, in July 2021, she was found to be neutropenic, with an ANC of 0.6 × 10^3^/L. Imatinib therapy was held and subsequently restarted the next month after the ANC returned to a normal range of 4.1 × 10^3^/L. However, imatinib was restarted at a decreased dose of 300 mg daily to prevent future recurrence of neutropenia. The following two months, the patient was found to be neutropenic again, with ANC values of 0.9 × 10^3^/L in September 2021 and 1.3 × 10^3^/L in October 2021. Dosage of imatinib was further reduced to 200 mg daily in hopes to prevent further neutropenic episodes. After this change, the patient had a stable ANC within the normal range for the following 10 months. Repeat labs in September 2022 showed an increase in BCR‐ABL1 transcript level to 36.629% from 11.152%, indicating poor treatment response. In response, her oncologic provider changed her imatinib regimen once again to be alternating days between 200 mg and 300 mg daily. When she was noted to be neutropenic again, in October 2022, with an ANC of 1.4 × 10^3^/L, and an additional increase in BCR‐ABL1 transcript level to 46.694% was observed, she was referred to the Medical University of South Carolina (MUSC) for a second opinion. The provider at MUSC suggested switching her from imatinib, which is a first‐generation TKI, to bosutinib 500 mg daily, a second‐generation TKI. This change, however, resulted in the patient having severe diarrhea, a known adverse effect with bosutinib. She was consequently switched to asciminib 80 mg daily, which is a different class of TKI inhibitors. Two months following this change in treatment, this patient’s BCR‐ABL1 transcript level was noted to be 1.001% in April 2023, the lowest it had been up until this point, indicating successful treatment. The patient, however, was still neutropenic, with an ANC of 1.2 × 10^3^/L. Despite the neutropenic lab findings, since the patient was asymptomatic, the decision was made to continue asciminib, given that she had excellent treatment response. The BCR‐ABL1 transcript level was consistently below 0.1%, which is the benchmark for successful treatment response. The patient continued to have a low normal ANC ranging from 1.5–1.8 × 10^3^/L but remained asymptomatic. In August of 2025, she was noted to be neutropenic again with an ANC of 1.2 × 10^3^/L. The persistence in neutropenia despite successful CML remission prompted her provider to check for Duffy antigen presence. Testing confirmed the patient lacked Fya and Fyb antigens, consistent with Duffy‐null status. Following identification of Duffy‐null status, the patient was continued on asciminib with reduced monitoring of her ANC given her asymptomatic presentation. Informed and written consent was obtained from this patient. A full timeline of this patient’s treatment and lab values is shown in Table 1.

**TABLE 1:** Dates and lab values corresponding to the patient’s CML therapy.

	Date	WBC	ANC	BCR‐ABL1 transcript level	Changes in therapy
Reference range	—	4.5–11 × 10^9^/L	1.5–8 × 10^3^/L	< 0.1%	—
	May 17, 2021	65	—	68.75%	—
	May 18, 2021	49.9	23.4	—	—
	June 14, 2021	15.6	9.8	—	Imatinib (Gleevec) 400 mg daily started
	July 15, 2021	2.1	0.6	—	Imatinib 400 mg on hold
	August 05, 2021	7.3	4.1	—	Imatinib 300 mg daily started (dose reduction)
	September 16, 2021	2.8	0.9	11.334%	—
	October 14, 2021	3.3	1.3	6.768%	—
	January 13, 2022	11.9	6.8	51.214%	Imatinib reduced to 200 mg daily (dose reduction)
	March 10, 2022	5.8	2.6	—	—
	April 07, 2022	4.2	1.7	—	—
	May 12, 2022	4.5	1.6	11.152%	—
	June 16, 2022	4.2	1.7	—	—
	July 18, 2022	4.5	2.1	—	—
	September 15, 2022	4.4	1.5	36.629%	—
	October 17, 2022	4.4	1.4	—	Dosing changed to 200 mg/300 mg on alternating days (dose increase)
	November 17, 2022	3.2	1.1	—	—
	December 01, 2022	3	1.1	46.694%	—
	December 15, 2022	3.1	0.9	—	Referral to MUSC
	January 11, 2023	3.7	1.3	9.978%	Started on bosutinib 500 mg daily
	February 13, 2023	4.6	2.1	—	Switched to asciminib 80 mg daily
	March 13, 2023	3.5	2.1	—	—
	April 08, 2023	3.6	1.2	1.001%	—
	May 15, 2023	4.4	1.5	—	—
	August 30, 2023	3.8	1.5	0.007%	—
	September 23, 2023	4.6	1.8	—	—
	November 29, 2023	4.2	1.8	0.012%	—
	April 01, 2024	3.7	1.6	0.013%	—
	July 22, 2024	4.7	1.8	0.014%	—
	October 21, 2024	4.2	1.7	—	—
	February 11, 2025	3.9	1.4	0.003%	—
	August 11, 2025	3.1	1.2	0.003%	—

## 3. Discussion

This case demonstrates the importance of identifying Duffy‐null individuals, especially since it is so common. This patient had multiple treatment dose reductions in response to recurrent neutropenia that may have contributed to her delayed remission of CML. In the treatment of CML, the goal of treatment is achieving major molecular response, which is BCR‐ABL1 transcript level < 0.1% by 12 months [6]. Despite achieving major molecular response in this patient for over 12 months, she had persistent neutropenia, leading to the investigation of Duffy‐null status. It is important to note that neutropenia is an expected adverse effect noted with imatinib therapy; however, her Duffy‐null status creates a unique clinical picture [7]. Recognition of this underlying phenotype is particularly important because an ANC that appears abnormal according to conventional reference ranges may not carry the same clinical significance in an otherwise asymptomatic Duffy‐null individual.

Treatment interruption and dose modification are established approaches for managing significant imatinib‐associated hematologic toxicity. Granulocyte colony‐stimulating factor (G‐CSF), also known as filgrastim, does have a role in recurrent or persistent TKI‐induced neutropenia to prevent therapy interruptions. However, CML requires continuous, long‐term TKI therapy. Although G‐CSF can be used as supportive therapy to temporarily increase the neutrophil count and may facilitate continuation of TKI therapy in selected patients, it does not address the underlying tendency toward TKI‐associated myelosuppression and therefore would not, by itself, provide a long‐term solution to recurrent neutropenia during ongoing TKI therapy. In retrospect, the later identification of Duffy‐null status provides additional context for her persistently lower ANC. There was no role for G‐CSF in this patient given her asymptomatic neutropenia and lack of Duffy antigens. Her clinical course suggests that TKI‐associated myelosuppression may have been superimposed upon a lower baseline neutrophil count associated with the Duffy‐null phenotype.

Neutropenia (ANC < 1.5 × 10^3^/L) is commonly below the threshold for participation in most clinical trials [8]. African American individuals make up the majority of people that have the Dully‐null phenotype; therefore, a large population of these individuals may be excluded from trials due to being Duffy‐null [4]. African American individuals are commonly underrepresented in clinical trials, leading to increased uncertainty in clinical decision‐making [9]. Unfortunately, there is no widely used reference range of normal for the ANC in Duffy‐null individuals. A study in 2023 aimed to develop a new ANC reference range for this population. The study suggested the use of the ANC range of 1.210 to 5.390 × 10^3^/L, which, if applied to our patient, meets criteria for a normal ANC more frequently than with standard reference ranges [10].

A case study published in 2025 reported a similar instance in an African American patient receiving chemotherapy for cervical cancer [3]. After having encountered neutropenia after three cycles of chemotherapy, she was found to be Duffy‐null, and the following chemotherapy cycles were administered through using a lowered neutropenic threshold of ANC < 1.0 rather than ANC < 1.5 [3]. The patient completed chemotherapy and has maintained remission. The discovery of this patient’s Duffy‐null status helped guide therapy and subsequent remission. Without this knowledge, there could have been treatment delays and chemotherapy dose reductions that may not have resulted in the favorable outcome that this patient had [3].

African American patients, in particular, would benefit from Duffy‐null testing prior to the initiation of cancer treatment. Duffy‐null status may be driving neutropenia and should be checked prior to reactively adjusting treatment. Identification of these individuals can help clinicians evaluate therapy status in a patient focused manner. In addition, development of a standardized Duffy‐null reference range can also prevent treatment delays and possible undertreatment. In this case, if Duffy status were known, therapy may not have been reduced in response to neutropenic episodes, given that Duffy‐null neutropenia is benign and the patient was asymptomatic [4]. This case study aims to contribute to existing literature about Duffy‐null status and encourages providers to investigate Duffy‐null status to improve disease outcomes.

## 4. Conclusion

This case presents a patient with recurrent neutropenic episodes, prolonging treatment and remission of CML. This case highlights the importance in identifying Duffy‐null individuals to prevent dose reductions in therapy and delaying remission in response to recurrent neutropenic episodes. This finding may benefit long‐term outcomes in the setting of cancer therapy. A future reference range for Duffy‐null‐associated neutropenia may help guide physicians in clinical judgment with these patients.

## Author Contributions

Anushka Parekh, DO: writing–original draft and writing–review and editing.

Joanna Metzner‐Sadurski, MD: supervision and writing–review and editing.

All authors contributed to the clinical management of the patient, data collection, and manuscript preparation.

## Funding

No funding was received for this work.

## Disclosure

All authors have read and approved the final version of the manuscript. The corresponding author, Anushka Parekh, DO, had full access to all of the data in this study and takes complete responsibility for the integrity of the data and the accuracy of the data analysis.

## Ethics Statement

The Edward Via College of Osteopathic Medicine Institutional Review Board does not require ethical approval of case reports that do not meet the definition of human subject research.

## Consent

Written informed consent was obtained from the patient. Completed consent form is available upon request.

## Conflicts of Interest

The authors declare no conflicts of interest.

## Supporting Information

Additional supporting information can be found online in the Supporting Information section.

## Supporting information


**Supporting Information 1** VCOM Blank Consent Document: blank consent form from Edward Via College of Osteopathic Medicine was provided. Informed consent received from the patient is available upon request.

**Supporting Information 2** CARE‐checklist.

## Data Availability

Data sharing is not applicable as no new data were generated, or the article describes entirely theoretical research.

## References

[1] Liu J. M. and Luo H. R. , Novel Neutrophil Biology Insights Underlying Atypical Chemokine receptor-1/Duffy Antigen Receptor of chemokines–associated Neutropenia, Current Opinion in Hematology. (2024) 31, no. 6, 302–306, 10.1097/MOH.0000000000000834. PMC11426986 39045882

[2] Legge S. E. , Christensen R. H. , Petersen L. et al., The Duffy-null Genotype and Risk of Infection, Human Molecular Genetics. (2020) 29, no. 20, 3341–3349, 10.1093/hmg/ddaa208. PMC7906776 32959868

[3] Kharel Z. , Rubin N. J. , David R. J. , and Kouides P. , Diagnosis of Duffy null–associated Neutropenia During Chemotherapy, American Journal of Hematology. (2025) 100, no. 6, 1105–1108, 10.1002/ajh.27679. 40186424

[4] Atallah-Yunes S. A. , Ready A. , and Newburger P. E. , Benign Ethnic Neutropenia, Blood Reviews. (2019) 37, 10.1016/j.blre.2019.06.003. PMC6702066 31255364

[5] National Cancer Institute , BCR::ABL1 Fusion Gene, NCI Dictionary of Cancer Terms, 2026, https://www.cancer.gov/publications/dictionaries/cancer-terms/def/bcrabl1-fusion-gene.

[6] Mohd Yacob A. , Muhamad N. A. , Chang K. M. , Akmal Hisham H. , Mat Yusoff Y. , and Ibrahim L. , Hsa-miR-181a-5p, hsa-miR-182-5p, and hsa-miR-26a-5p as Potential Biomarkers for BCR-ABL1 Among Adults with Chronic Myeloid Leukemia Treated with Tyrosine Kinase Inhibitors at the Molecular Response, BMC Cancer. (2022) 22, no. 1, 10.1186/s12885-022-09396-5. PMC8962036 35346116

[7] Novartis Pharmaceuticals Corporation , Gleevec (imatinib mesylate) tablets [Prescribing information], 2008, U.S. Food and Drug Administration, https://www.accessdata.fda.gov/drugsatfda_docs/label/2008/021588s024lbl.pdf.

[8] Abdallah M. , Arters F. , Patel J. et al., When a Number is Just a Number: Duffy-Null Blood Group Phenotype, Absolute Neutrophil Count, and Incidence of Neutropenic Fever and Chemotherapy Dose Reductions in Patients Treated for Plasma Cell Dyscrasias [Abstract], Blood. (2023) 142, no. S1, 10.1182/blood-2023-187811.

[9] Lynce F. , Blackburn M. J. , Zhuo R. et al., Hematologic Safety of Palbociclib in Combination with Endocrine Therapy in Patients with Benign Ethnic Neutropenia and Advanced Breast Cancer, Cancer. (2021) 127, no. 19, 3622–3630, 10.1002/cncr.33620. PMC11864073 34157782

[10] Merz L. E. , Osei M. A. , Story C. M. et al., Development of Duffy Null–specific Absolute Neutrophil Count Reference Ranges, JAMA. (2023) 329, no. 23, 2088–2089, 10.1001/jama.2023.7467. PMC10282887 37338884

