# Supplementary material for: A Case of Duffy‐Null‐Associated Neutropenia in a Patient With Chronic Myeloid Leukemia
Source: Case Rep Hematol. 2026 Sep 26;2026:9335086. doi: 10.1155/crh/9335086 (PMC13615517; doi:10.1155/crh/9335086)
Supplement: Supplementary file 2 [file CRH-2026-9335086-s002.docx]

Edward Via College of Osteopathic Medicine

Consent Form for Case Reports

**For a patient’s consent to publication of images and/or information
about them in journals or other publications.**

| Name of person described in article or shown in photograph: |  |  |
| --- | --- | --- |
| Description of photo, image, text or other material (the Information) about the patient: |  |  |
|  |  |  |
| Provisional title of article in which material will be included: |  |  |
| Name of medical practitioner or corresponding author: |  |  |

I [print full name] _________________________, give my consent for this information about me or my child/ward or relative [print full name], ___________________, relating to the subject matter above (“the Information”) to appear in a journal article, or to be used for the purpose of a presentation.

I understand the following:

1. The Information will be published without my name/child’s name/relative’s name attached and every attempt will be made to ensure anonymity. I understand, however, that complete anonymity cannot be guaranteed. It is possible that somebody somewhere - perhaps, for example, somebody who looked after me/my child/relative, if I was in hospital, or a relative - may identify me.
2. The Information may be published in a journal which is read worldwide or an online journal. Journals are aimed mainly at health care professionals but may be seen by many non-doctors, including journalists.
3. The Information may be placed on a website.
4. I/my child/my relative will not receive any financial benefit from publication of the article.
5. I can withdraw my consent at any time before online publication, but once the Information has been committed to publication it will not be possible to withdraw the consent.

Signed: __________________________________	Date: ______________________

Signature of person who has explained and administered the form to the patient or their representative (requesting medical practitioner or corresponding author):

________________________________________	Date:______________________
